# Combined Potential of Orlistat with Natural Sources and Their Bioactive Compounds Against Obesity: A Review

**DOI:** 10.3390/molecules30112392

**Published:** 2025-05-30

**Authors:** Jonatan Jafet Uuh Narvaez, Ivan Chan Zapata, Maira Rubi Segura Campos

**Affiliations:** 1 Facultad de Ingeniería Química, Universidad Autónoma de Yucatán, Periférico Norte, Kilómetro 33.5, Tablaje Catastral 13615, Chuburná de Hidalgo Inn, Mérida 97203, Yucatán, Mexico; jonatan.uuh@correo.uady.mx (J.J.U.N.); ivanchz1990@gmail.com (I.C.Z.); 2 Centro de Investigación Científica de Yucatán, Calle 43 No. 130 x 32 y 34, Chuburná de Hidalgo, Mérida 97205, Yucatán, Mexico

**Keywords:** dyslipidemia, pancreatic lipase inhibition, combinatorial therapy, extracts

## Abstract

Obesity represents a significant global public health issue, contributing to the rising prevalence of metabolic diseases. One treatment for obesity is orlistat, a drug that inhibits pancreatic lipase. It is widely used due to its efficacy in reducing dietary fat absorption. However, patient adherence to this drug is often hindered by its associated adverse effects. As a result, there is an increasing interest in exploring alternative therapeutic options derived from natural sources, such as plants and algae, particularly extracts and their bioactive compounds. These extracts and compounds have shown potential in inhibiting pancreatic lipase and other markers associated with obesity. Nevertheless, they also present certain limitations, including low bioavailability. In this context, combination therapy involving orlistat and these extracts or their compounds has emerged as a promising strategy. This approach aims to enhance the inhibition of pancreatic lipase and other obesity-related markers, thereby improving therapeutic outcomes and reducing adverse effects associated with treatment. The objective of this review is to analyze the available scientific evidence regarding the combined effects of orlistat and extracts or bioactive compounds in inhibiting various markers related to dyslipidemia and obesity, with the goal of proposing combination therapy as a safe and effective therapeutic option.

## 1. Introduction

Obesity is a major public health crisis worldwide, with significant implications for the incidence of metabolic, cardiovascular, and neoplastic diseases. According to the World Health Organization (WHO), in 2022, approximately 2.5 billion adults (43% of the world’s population) were overweight, and among them, 890 million (16%) were classified as obese. Projections indicate that by 2035, more than 4 billion people (51% of the global population) could be overweight or obese [1].

Obesity arises from a chronic imbalance between caloric intake and energy expenditure, resulting in the excessive accumulation of adipose tissue. This imbalance disrupts energy homeostasis, thereby promoting a pro-inflammatory environment, insulin resistance, and alterations in endocrine signaling. Collectively, these factors increase the risk of cardiovascular and metabolic complications [2,3].

During digestion, pancreatic lipase plays a central role in the absorption of lipids. It catalyzes the hydrolysis of triglycerides (TG) into free fatty acids and monoglycerides, thereby facilitating their subsequent absorption in the small intestine. Therefore, modulating this enzyme is imperative for reducing caloric absorption from lipids, establishing it as an important therapeutic target in the treatment of obesity [4,5].

Orlistat, a drug derived from lipstatin, is a compound that was originally isolated from the cultures of *Streptomyces toxytricini*. Its lipid-like structure enables specific binding to the active site of pancreatic lipase [6]. This action prevents the hydrolysis of TG, reducing fat absorption in the intestine and, consequently, total caloric intake. However, the therapeutic benefit of orlistat is limited by gastrointestinal side effects, such as steatorrhea, flatulence, diarrhea, and abdominal discomfort, which result from the presence of undigested lipids in the intestinal tract. These complications have the potential to adversely affect adherence to treatment and the quality of life of patients [4,7].

Recent research into bioactive compounds and extracts has revealed their promising potential to influence lipid metabolism. For instance, bioactive compounds such as polyphenols have demonstrated effective inhibition of pancreatic lipase, highlighting their potential role in natural therapies for obesity and other metabolic disorders [4,8]. However, low bioavailability of the compounds in the gastrointestinal tract limits their pharmacological efficacy [9]. While studies have suggested that strategies such as nanoencapsulation and the use of lipid transporters can protect bioactive compounds during digestion and improve absorption, further research is still required for their application [10].

Consequently, alternative strategies that may complement the use of orlistat are needed. Combination therapy with orlistat could represent an alternative strategy to enhance the control of lipid metabolism. The co-administration of lipase inhibitors with bioactive compounds could create a synergistic effect that optimizes the inhibition of fat digestion and absorption while minimizing the adverse effects associated with orlistat [11,12]. Nevertheless, the safety and efficacy of these combinations must be adequately evaluated, as interactions between compounds can result in unexpected positive and negative consequences [13]. The aim of this review was to analyze the combined effects of orlistat, along with extracts or other natural-derived materials and their bioactive compounds, on pancreatic lipase inhibition, in vivo models, and clinical trials to identify more effective and safer therapeutic strategies for the treatment of obesity.

## 2. Lipid Uptake: A Therapeutic Target in the Management of Obesity

Obesity is a chronic metabolic disease characterized by excessive accumulation of adipose tissue, resulting from a sustained imbalance between energy intake and metabolic expenditure [2,3]. A critical period in maintaining energy homeostasis is the postprandial phase, during which lipids are processed and absorbed [14].

During this phase, pancreatic lipase (composed of 449 amino acids) employs a catalytic triad (Ser152, Asp176, and His263) to digest lipids, converting TG into fatty acids and monoglycerides [15]. Initially, TG are emulsified by bile acids, allowing their dispersion into finely particulate droplets and facilitating the action of pancreatic lipase [16]. The activation of this enzyme, initiated by colipase, leads to the hydrolysis of TG into free fatty acids and monoglycerides. These components are subsequently integrated into nanometer-sized micelles, which are then transported to the apical membrane of enterocytes [17]. Subsequent to the process of absorption, lipids are re-esterified and packaged into chylomicrons. These chylomicrons enter the lymphatic system and, thereafter, the bloodstream [16].

The postprandial absorption process is essential for energy production, regulation of lipid metabolism, and endocrine signaling. However, an acute elevation of postprandial TG can trigger a series of detrimental effects on endothelial function, compromising vasodilatory capacity and promoting a cascade of proinflammatory, pro-thrombotic, and atherogenic responses [16,18]. A notable increase in chylomicrons and other lipoproteins following the ingestion of high-fat foods has been associated with a significant rise in oxidative stress and systemic inflammatory activity. The observed increase in inflammation activates signaling pathways, including nuclear factor κB (NF-κB), which in turn leads to the generation of reactive oxygen species, compromised vascular integrity, and the facilitation of the formation of atherosclerotic plaques [19,20]. These adverse effects underscore the importance of maintaining plasma TG levels within a specific range (>2.48 mmol/L) during the initial two postprandial hours to mitigate the risk of these complications [21,22]. Consequently, postprandial hyperlipidemia emerges as a significant therapeutic target in the management of obesity.

In the therapeutic approach to obesity, the effectiveness of intensive diet and exercise interventions is often limited by a lack of sustained adherence. Studies indicate that long-term programs have significant dropout rates. A meta-analysis of 14 clinical trials conducted between 2012 and 2023 revealed that short-term lifestyle interventions (≤3 months) exhibited a retention rate of approximately 80% among participants. Conversely, in the context of longer programs (3–6 months), the retention rate declined to approximately 70%, suggesting that longer programs face higher dropout rates (approximately 30% at 6 months) [23]. Research indicates that adherence to dietary recommendations is less than 50% over an extended period. Among individuals who initiate medication, adherence to treatment varies according to the drug. Retrospective data from 1911 patients (2015–2022) revealed that the average 12-month persistence rate with any anti-obesity medication was only 19% [24].

The classification of drugs employed in the treatment of obesity is based on their respective mechanisms of action. Lipid-lowering agents, such as statins, are compounds that reduce the synthesis of hepatic cholesterol [25]. Fibrates have been demonstrated to augment fatty acid oxidation through the activation of peroxisome proliferator-activated receptor (PPAR) α [26]. In conjunction with cholesterol absorption inhibitors, bile acid sequestrants have been shown to decrease the recirculation and formation of lipoproteins [27]. Finally, inhibitors of intestinal lipid absorption, such as orlistat, act by blocking the action of pancreatic lipase, thereby decreasing the digestion of TG and limiting the absorption of fatty acids in the intestine [28].

## 3. Orlistat: A Pancreatic Lipase Inhibitor Drug

Pharmacological interventions represent a complementary strategy in the management of obesity, particularly for patients who have not achieved sustained weight loss through lifestyle modifications alone, such as dietary changes and increased physical activity [29]. Among the primary pharmaceutical therapies available, orlistat has been in use since its approval in 1998 [30].

Orlistat (tetrahydrolipstatin, C_29_H_53_NO_5_) is a compound characterized by a chemical structure that includes a 2,3-disubstituted β-lactone, a long-saturated acyl chain, and an N-formyl-L-leucine residue linked by an ester bond. It is a derivative of lipstatin, a metabolite produced by *S. toxytricini* through a fermentation process, which is then transformed into orlistat via catalytic hydrogenation [31].

**Figure 1:**
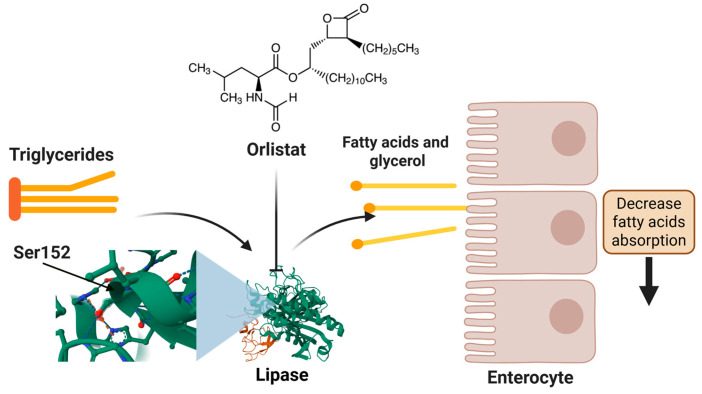
Orlistat’s mechanism of action in lipid regulation.

The mechanism of action of orlistat involves the inhibition of pancreatic and gastric lipase, thereby preventing the hydrolysis of dietary TG into absorbable free fatty acids. This results in a reduction of approximately 30% in fat absorption (Figure 1). Orlistat’s action occurs within the intestinal lumen, with minimal systemic absorption (<2%), thereby circumventing the potential for cardiovascular or neuropsychiatric adverse effects [32,33,34].

Molecular docking studies demonstrate that orlistat fits into the active site cavity of the enzyme when the lid is in the open conformation. The drug’s β-lactone ring forms a covalent bond with Ser152, while its hydrophobic aliphatic chains occupy the lid region and the adjacent hydrophobic pocket, establishing interactions with key residues such as Gly76-Phe80 and Leu213-Met217 of pancreatic lipase. The inhibition exhibited by orlistat is competitive in nature, with reported mean inhibitory concentrations (IC_50_) ranging from 0.1 to 0.2 μM, as documented in various studies [35].

In clinical trials, the administration of orlistat has demonstrated a reduction in the area under the curve (AUC) of postprandial TG of approximately 17% compared to placebo, thereby supporting its effect on modulating lipid metabolism [36]. The pharmaceutical agent is generally administered at a dose of 120 mg thrice daily with meals, and patients are advised to adhere to a moderate-calorie, low-fat diet to optimize efficacy and mitigate gastrointestinal adverse effects [33,37]. In a randomized trial lasting between 6 and 12 months, orlistat in conjunction with a low-calorie diet led to a mean additional weight reduction of 2.5 to 3 kg compared to the placebo group [38]. Furthermore, its beneficial effects are linked to enhancements in various parameters, including decreases in waist circumference (WC), blood pressure (BP), glucose levels, and blood lipids [39].

Despite its efficacy, orlistat is associated with adverse effects, primarily in the gastrointestinal tract, due to unabsorbed fat remaining in the intestinal lumen. The most common symptoms include abdominal distension and pain, steatorrhea (fatty and bulky stools), flatulence with oily discharge, urgency to defecate, fecal incontinence, increased bowel frequency, and diarrhea [30]. Due to its mechanism of action, orlistat also has the potential to reduce the absorption of fat-soluble vitamins (A, D, E, and K) and β-carotenoids. This may have implications for the nutritional status of individuals undergoing prolonged treatment regimens [40]. Consequently, there is an urgent need for effective and safe alternative anti-obesity therapies.

## 4. Natural Sources and Their Bioactive Compounds as a Proposal Against Postprandial Hypertriglyceridemia

Bioactive compounds are defined as substances found in various natural sources that have demonstrated biological effects on human health, contributing to the prevention of chronic diseases when consumed regularly [41]. Among the main bioactive compounds are plant-derived secondary metabolites, including polyphenols, flavonoids, tannins, and alkaloids, which are present in various foods and medicinal plants. While secondary metabolites have been demonstrated to play a role in ecological mechanisms such as defense, competition, or attraction, they have also been shown to have multiple benefits for human health [42]. Several review articles have thoroughly documented the effects of plant extracts and their bioactive compounds as proposed anti-obesity therapies through various mechanisms of action. These mechanisms include the inhibition of adipocyte differentiation, the browning of white adipose tissue, suppression of inflammation, improvement of gut microbiota, reduction of obesity-inducing genes, and inhibition of pancreatic lipase [43,44,45].

Previous research has documented the inhibition of pancreatic lipase by 93 species from 48 distinct plant families, in addition to a considerable number of bioactive compounds. The Fabaceae and Lamiaceae families showed the greatest abundance, with seven species demonstrating inhibitory effects of up to 95% on pancreatic lipase activity [4]. Moreover, clinical trials have yielded evidence concerning the effects of polyphenol-rich foods administered with fatty meals on postprandial lipemia. A recent study involving 37 healthy adults revealed that the administration of a *Chrysanthemum morifolium* extract (containing flavonoid glycosides and cynaroside > 30%) with a high-fat meal led to a significant reduction in postprandial TG levels compared to a control group [46]. Similarly, a pilot clinical trial reported that the addition of spice blends (cinnamon, turmeric, oregano, rosemary, and others) to high-fat meals resulted in a substantial decrease in postprandial TG levels (18 mg/dL) compared to unseasoned meals [47].

Nevertheless, a fundamental constraint on the efficacy of bioactive compounds pertains to their bioavailability and pharmacokinetics. A significant number of polyphenols demonstrate limited intestinal absorption and undergo substantial metabolic transformation through phase II reactions (conjugation) or via the intestinal microbiota prior to reaching systemic circulation [48,49]. For instance, anthocyanins have demonstrated limited bioavailability and predominantly circulate as degraded phenolic metabolites [50]. However, the observed clinical effects suggest that efficacy may be mediated by these circulating metabolites or by direct local actions in the gastrointestinal tract, such as modulation of the intestinal microbiota [48,51].

Therefore, it is imperative to explore strategies that enhance the bioavailability and stability of bioactive compounds during gastrointestinal transit, with the aim of optimizing their therapeutic efficacy in the management of postprandial hyperlipidemia. Such strategies may include encapsulation, the implementation of controlled-release systems, or combination therapies with other compounds (or drugs) that enhance absorption and biological activity, thereby amplifying their potential as alternative treatments or adjuncts for health [13,52,53].

## 5. Interactions Between Orlistat and Bioactive Compounds

Extensive research has been conducted on combinatorial therapies that integrate extracts from natural sources or their bioactive compounds with conventional drugs for the treatment of various diseases, including diabetes, hypertension, and obesity [13,54,55]. This therapeutic approach aims to reduce drug dosages, thereby minimizing the occurrence of adverse effects [56]. Nevertheless, concerns have been raised regarding the indiscriminate use of extracts in conjunction with drugs, as this combination can either enhance or impair the efficacy of pharmaceutical treatments [57].

In the context of combinatorial therapy involving an extract or bioactive compound, interactions can generate a variety of effects, including synergy, additivity, or antagonism. A synergistic effect occurs when the combination produces a benefit that exceeds the expected sum of the individual effects. An additive effect is approximately equivalent to the direct sum of these effects, with no observable increase or decrease. Antagonism occurs when the combination results in a reduction in efficacy compared to the individual effects of the compounds [58]. The classification of these effects can be facilitated by employing the combination index (CI). A CI less than 1 signifies synergy, while a CI equal to 1 indicates additivity. Conversely, a CI greater than 1 indicates antagonism [59]. Consequently, it is imperative to investigate the interactions between orlistat and bioactive compounds to enhance therapeutic efficacy and ensure patient safety.

Inhibition mechanisms have the potential to enhance our understanding and facilitate prediction of the outcomes of combinations of orlistat with bioactive compounds, including synergistic, additive, or antagonistic effects [13]. Orlistat has been shown to bind covalently and irreversibly to the Ser152 residue in the active site of pancreatic lipase, thereby causing a permanent reduction in Vmax for TG hydrolysis [40]. While non-competitive inhibitors have been demonstrated to exhibit synergy when employed in conjunction with orlistat, competitive inhibitors frequently yield additive or antagonistic effects. However, the precise nature of each inhibition mechanism remains to be elucidated in regard to its influence on the magnitude and type of combined response. For example, the flavonoid kaempferol competes with the substrate at the active site, elevating Km and demonstrating synergy (CI < 1) when paired with orlistat [11]. In contrast, epigallocatechin gallate exhibits allosteric binding via hydrogen bonds with Val21, Glu188, and Glu220, leading to a reduction in Vmax without altering Km. This mechanism potentiates the combined effect [12,60]. Furthermore, additional characterization of mixed-type inhibitors and their potential combinatorial effects with orlistat is necessary.

From an efficacy perspective, identifying synergistic combinations facilitates the development of more effective therapeutic interventions. These combinations have the potential to mitigate the toxicity and adverse effects associated with the administration of high doses of individual drugs. This allows for the inhibition of biological compensation mechanisms, optimization of the dosage of each compound, and activation of multiple therapeutic targets. From a safety perspective, the absence of evaluation of a combination could lead to antagonistic interactions that diminish treatment efficacy or augment toxicity [58]. Consequently, a better understanding of these interactions is essential to propose combinatorial therapy as a safe and effective therapeutic alternative.

## 6. Natural Sources and Their Anti-Obesity Effects in Combination with Orlistat

Various research groups have dedicated themselves to investigating the potential of extracts and other materials from natural sources as anti-obesity agents, given their low to non-toxic characteristics. These investigations have focused on several medicinal and edible plants, along with other natural foods such as seaweed. The findings of these studies suggest that the mechanisms underlying the anti-obesity effects of these species may involve the reduction of lipid and carbohydrate absorption, regulation of lipid metabolism, and other processes, as observed in different in vitro and animal models. Furthermore, these extracts and natural-derived materials have been demonstrated to exert beneficial effects on patients [61,62].

The present review focuses on the anti-obesity effects of extracts and other materials from natural sources in conjunction with orlistat. Six studies examined the effects of plant extracts, fractions, and other natural-derived materials (capsules or psyllium), while one study evaluated the properties of a mixture of plant aqueous distillates. Furthermore, two studies examined the in vitro and in vivo anti-obesity potential of seaweed extracts and fractions. The results of the aforementioned studies are summarized in Table 1, which details the biological models used, the effects observed, and the types of interactions. In several studies, it was found that the combination of extract/fraction with orlistat exhibited greater anti-obesity efficacy compared to the individual extract/fraction or orlistat.

The most extensively studied approach in the search for novel anti-obesity agents focuses on the inhibition of pancreatic lipase activity, due to the scarcity of compounds that directly interact with this enzyme. Pancreatic lipase plays a pivotal role in the absorption of dietary TG through the hydrolysis of TG into monoglycerides and fatty acids, as previously described. Consequently, pancreatic lipase inhibitors reduce the activity of the enzyme, primarily attenuating fat absorption [71,72].

In this context, the potential benefits of combining certain extracts with orlistat have been explored. A previous study demonstrated that various combinations of orlistat with the methanol extract of leaves from *Camellia sinensis* (L.) Kuntze (tea plant) or two derived fractions (caffeine- and polyphenol-rich fractions) exhibited a synergistic effect on the inhibition of pancreatic lipase. The CI values were less than 1 (CI = 0.35–0.93) and the IC_50_ values ranged from 3.48 to 7.73 µg/mL. These IC_50_ values were found to be lower than those of the extract and fractions evaluated individually (IC_50_ = 16.96–35.73 µg/mL). Subsequent fractionation of the polyphenol-rich fraction through column chromatography yielded eight fractions, each demonstrating a pancreatic lipase inhibitory synergistic effect in combination with orlistat (IC_50_ = 1.93–11.95 µg/mL and CI = 0.47–0.99). Furthermore, these IC_50_ values were found to be lower than those observed for the fractions evaluated individually (IC_50_ = 10.06–40.67 µg/mL). A high-performance liquid chromatography (HPLC) analysis was conducted to identify the major compounds present in the synergistically active fractions. The analysis revealed the presence of epicatechin gallate and epigallocatechin gallate. The synergistic inhibitory effects of these compounds on pancreatic lipase when combined with orlistat are described below [12].

Conversely, there is evidence that the combination of orlistat with other natural-derived materials does not result in a synergistic effect on the inhibition of pancreatic lipase. A tannin-rich fraction of the brown edible seaweed *Ascophyllum nodosum* (L.) Le Jolis has been shown to inhibit pancreatic lipase activity (49.5% activity). However, when this fraction was combined with orlistat (which demonstrated 38.6% inhibition), no significant inhibitory effect was observed (47.0% activity). The authors suggested that the compounds present in the tannin-rich fraction may bind to the active site, thereby exhibiting an antagonistic effect. Furthermore, the researchers hypothesized that these compounds might bind to an alternative site on pancreatic lipase, altering its active site topology and interfering with the effect of orlistat [63].

In addition to in vitro tests with pancreatic lipase, preclinical animal models of obesity have emerged as a significant tool for evaluating the potential effects of extracts and other natural-derived materials. Mice and rats are the most frequently utilized rodent models in in vivo assays due to their anatomical and physiological similarities to humans. These models enable the screening of new alternative sources with anti-obesity properties prior to their application in human studies [73]. Consequently, numerous studies have utilized these models to elucidate the beneficial effects of combinations of natural-derived materials and orlistat.

The synergistic inhibitory effects of an aqueous extract from *Elettaria cardamomum* (cardamom, 500 mg/kg) in combination with orlistat (10 mg/kg) were evaluated in C57BL/6 mice with induced obesity. The combination of the extract and the drug resulted in a significant decrease in body weight (BW), Lee obesity index (LOI, an indicator of obesity), and blood glucose levels compared to a control group of animals treated with orlistat alone. Furthermore, the combination of *E. cardamomum* extract and orlistat resulted in a reduction of low-density lipoprotein (LDL) cholesterol (89 mg/dL), total cholesterol (TC, 83 mg/dL), and TG (61.81 mg/dL), as well as in the atherogenic index (AI, 0.33) and cardiac risk ratio (CRR, 0.29). Some of these values were lower in comparison to those observed in the control group (LDL cholesterol = 111.027 mg/dL, TC = 100.40 mg/dL, TG = 60.81 mg/dL, AI = 0.42, and CRR = 0.43). The authors of this study also ascertained that the combination of *E. cardamomum* extract and orlistat produces beneficial effects on neuroinflammation and memory associated with obesity [64].

In another study, it was demonstrated that *Gelidium elegans*, a type of edible seaweed, possesses properties that enhance the effectiveness of orlistat. In this research, the *G. elegans* extract (50 mg/kg) combined with orlistat (20 mg/kg) led to a reduction in BW gain and the percentage of BW (subcutaneous fat) in a model of ICR mice with induced obesity. The observed reduction was significantly different from that of the control group treated with the commercial pancreatic lipase inhibitor alone. The authors also noted that the mixture of extract and orlistat resulted in increased serum high-density lipoprotein (HDL) cholesterol levels (109.6 mg/dL), which were higher compared to the orlistat group (88.6 mg/dL). Furthermore, the combination of extract and orlistat led to a decrease in serum TG (<60 mg/dL), hepatic TG (<125 mg/g liver), serum insulin (<5 ng/mL), and blood glucose (116.7–156.0 mg/dL). These values were lower than those observed in the control group (serum TG > 120 mg/dL, hepatic TG < 150 mg/g liver, serum insulin > 5 ng/mL, and blood glucose = 122.0–169.8 mg/dL) [65].

In order to comprehend the mechanism underlying the synergistic effect of the *G. elegans* extract and orlistat, the researchers assessed the expression of markers associated with adipose tissue and hepatic lipogenesis. The combination of extract and orlistat resulted in a significant increase in adenosine monophosphate-activated protein kinase (AMPK) phosphorylation (>175%) and protein expression of PR domain-containing16 (PRDM16, >130%) in hepatic tissue. In addition, a decrease in the protein expression of sterol regulatory element binding protein-1 (SREBP-1, <80%), acetyl-CoA carboxylase (ACC, <40%), diacylglycerol O-acyltransferase-1 (DGAT-1, <60%), and fatty acid synthase (FAS, <30%) was observed. The combination of extract and orlistat also produced a decrease in the protein expression of CCAAT/enhancer binding protein α (C/EBPα, <90%) and PPARγ (<90%) in white adipose tissue. The observed percentages were higher (protein expression of AMPK < 160% and PRDM16 < 120%) and lower (protein expression of C/EBPα > 100%, PPARγ > 90%, SREBP-1 > 60%, ACC > 60%, DGAT-1 > 60%, and FAS > 30%) than those observed in the orlistat control group. An increase in AMPK phosphorylation (>225%) and the protein expression of PRDM16 (>150%) and uncoupling protein-1 (UCP-1, >130%) was observed in the brown adipose tissue of the ICR mice treated with the *G. elegans* extract and orlistat. The observed values were also higher than those in the control group (protein expression of AMPK < 200%, PRDM16 < 200%, and UCP-1 < 140%). Finally, the administration of the extract and orlistat exhibited a synergistic effect in reducing hepatic lipogenesis, as determined by histopathological assays. This reduction was further substantiated by changes in liver weights (1.4 g in the combination group versus 1.6 g in the orlistat group) [65].

On the other hand, recent studies have shown positive effects when combining various extracts and orlistat in other in vivo models, such as rats. In research involving Wistar rats with induced obesity, the potential health benefits of an ethanol extract from leaves of *Hibiscus tiliaceus* L. (commonly known as sea hibiscus, 250 mg/kg) in combination with orlistat (10 mg/kg) were assessed. The treatment with the extract and orlistat resulted in a reduction in BW (174.44 g), which was lower compared to the control group treated with orlistat alone (221.39 g). The combination also led to decreases in the serum levels of TG (166.92 mg/dL) and TC (188.33 mg/dL), while increasing HDL cholesterol levels (67.84 mg/dL). These values were comparable to those observed in the orlistat group (TG = 164.85 mg/dL, TC = 191.28 mg/dL, and HDL cholesterol = 70.70 mg/dL), and statistically significant compared to the levels of TG (219.18 mg/dL), TC (233.97 mg/dL), and HDL cholesterol (33.94 mg/dL) in the control group without treatment [66].

*Allium sativum* (garlic) is another plant that has been demonstrated to possess anti-obesity activity when combined with orlistat, according to recent research conducted on Sprague-Dawley rats with induced obesity. In this study, an aqueous extract of cloves from *A. sativum* (500 mg/kg) was administered in conjunction with orlistat (30 mg/kg). This treatment resulted in a decrease in feed intake (<150 g) compared to the control group treated with orlistat (>150 g). Regarding BW, the combination of *A. sativum* extract and orlistat led to a decrease in BW, with values approaching those observed in groups treated with the individual extract or orlistat (BW = 250–300 g). Furthermore, the combination resulted in reductions in the levels of free fatty acids (<100 µmol/L), glucose (<125 mg/dL), insulin (<100 µIU/mL), homeostasis model assessment of insulin resistance (HOMA-IR) index (<20), and leptin (<4 ng/mL), while an increase in ghrelin (>0.3 Pg/mL) and adiponectin (>20) levels was noted. These values differed from those of the groups treated with only orlistat or the extract [67].

In addition to evaluating individual plants, there is evidence of the properties of medicinal plant mixtures in combination with orlistat on in vivo models. In traditional medicine, Arq-Kasni, Arq-Badiyan, and Arq-Makoh, which are aqueous distillates derived from *Cichorium intybus* L. (chicory), *Foeniculum vulgare* Mill. (fennel), and *Solanum nigrum* L. (black nightshade), respectively, have been used for the management of dyslipidemia and obesity. The anti-obesity effects of the mixture of these distillates (10 mL/kg/each distillate) and orlistat (5 mg/kg) were tested in Sprague-Dawley rats with induced dyslipidemia and obesity. The combination of the distillates and orlistat led to a reduction in BW gain, food efficiency ratio (FER), body mass index (BMI), heart weight-to-body weight (HW:BW) ratio, and relative liver weight (RLW). These values (<30 g, <0.075, <0.75 gm/cm^2^, <4, and <7.5, respectively) were lower than those of the control group treated with orlistat alone (BW > 30 g, FER > 0.10, BMI > 0.75 gm/cm^2^, HW:BW ratio > 4, and RLW > 6). Likewise, the levels of glucose, insulin, and leptin were lower in the group of animals treated with the three distillates and orlistat (glucose < 125 mg/dL, insulin < 1.25 µg/mL, and leptin < 2.5 ng/mL) compared to the control group (glucose > 125 mg/dL, insulin > 1 µg/mL, and leptin > 2.5 ng/mL) [68].

The authors also evaluated whether the distillates from *C. intybus*, *F. vulgare*, and *S. nigrum* combined with orlistat would alter the lipid profile of the rats. The observed outcomes following the administration of the mixture of distillates and the commercially available pancreatic lipase inhibitor included an increase in HDL cholesterol (>30 mg/dL) and a decrease in the serum levels of TC (<110 mg/dL), TG (<110 mg/dL), and LDL cholesterol (<75 mg/dL). In a similar manner, the combination of the three distillates and orlistat led to a reduction in AI (<3) and the serum levels of lipase (<200 U/L), lactate dehydrogenase (LDH, <125 IU/L), and thiobarbituric acid reactive substances (TBARS, <75 nmol/g tissue protein). Concurrently, there was an increase in the levels of superoxide dismutase (SOD) (>8 Units/mg protein). These values were found to differ from those observed in the orlistat group. Finally, histopathological assays revealed that the liver and heart of the animals treated with the distillates in conjunction with orlistat exhibited normal cellular morphology. In contrast, the orlistat group demonstrated mild liver steatosis, accompanied by slight deterioration of liver architecture and mild cellular changes in the heart without collagen deposition [68].

In addition to in vitro and in vivo tests, the implementation of clinical trials is imperative to ascertain the safety and anti-obesity effectiveness of bioactive compounds on human health. Numerous clinical studies have demonstrated the efficacy of various natural sources in the prevention and management of obesity and its complications [74,75]. Nevertheless, the current evidence concerning the anti-obesity properties of these natural sources in combination with orlistat is limited.

*Garcinia cambogia*, also known as Malabar tamarind, has been shown to play an important role in weight reduction. This has led to the evaluation of its anti-obesity potential when used in combination with orlistat. The study participants were obese patients administered *G. cambogia* capsules (166 mg/day) in conjunction with orlistat (120 mg/day). Subsequently, the anthropometric and cardio-metabolic parameters of the participants were assessed. The results revealed a decrease in BW (83.22 kg), BMI (30.45 kg/m^2^), WC (84.16 cm), hip circumference (HC, 102.17 cm), visceral adiposity index (VAI, 2.17), systolic BP (SBP, 133.10 mmHg), pulse pressure (PP, 52.97 mmHg), and mean BP (MBP, 106.61 mmHg) in patients treated with the mixture of *G. cambogia* and the commercial pancreatic lipase inhibitor. In contrast, the group treated with orlistat alone exhibited significantly higher values (BW = 90.55 kg, BMI = 36.50 kg/m^2^, WC = 91.76 cm, HC = 110.88 cm, VAI = 3.32, SBP = 143.12 mmHg, PP = 62.45 mmHg, and MBP = 111.89 mmHg) [69].

In the aforementioned study, the authors investigated the effects of the combination of *G. cambogia* and orlistat on the lipid profile of obese patients. The mixture resulted in a reduction of TG levels (221.92 mg/dL), TC (187.31 mg/dL), very low-density lipoprotein (VLDL) cholesterol (44.38 mg/dL), and CRR (3.81), while concurrently increasing HDL cholesterol (49.11 mg/dL). Furthermore, a decrease in postprandial blood glucose levels was observed (131.17 mg/dL). In contrast, the values observed in the orlistat group were significantly different (TG = 320.69 mg/dL, TC = 200.77 mg/dL, VLDL cholesterol = 64.13 mg/dL, CRR = 4.46, HDL cholesterol = 44.99 mg/dL, and postprandial blood glucose = 139.95 mg/dL). The researchers proposed a plausible additive effect of *G. cambogia* and orlistat on weight reduction in obese patients [69].

Finally, a clinical study has determined that the combination of psyllium (a fiber derived from the seeds of *Plantago ovata*, commonly known as blonde psyllium) and orlistat can mitigate the adverse effects of the latter drug. In this context, obese patients were treated with a mixture of psyllium (6.0 g) and orlistat (120 mg), resulting in a decrease in BW (94.96 kg) and BMI (37.40 kg/m^2^). However, patients treated with only orlistat also exhibited a reduction in BW (96.71 kg) and BMI (36.9 kg/m^2^). The combination of psyllium and orlistat demonstrated a significant reduction in gastrointestinal events (oily spotting = 42, fecal urgency = 41, and incontinence = 11) during the trial when compared to the group of subjects treated with orlistat alone (oily spotting = 249, fecal urgency = 132, and incontinence = 22) [70].

## 7. Bioactive Compounds from Natural Sources and Their Anti-Obesity Effects in Combination with Orlistat

**Figure 2:**
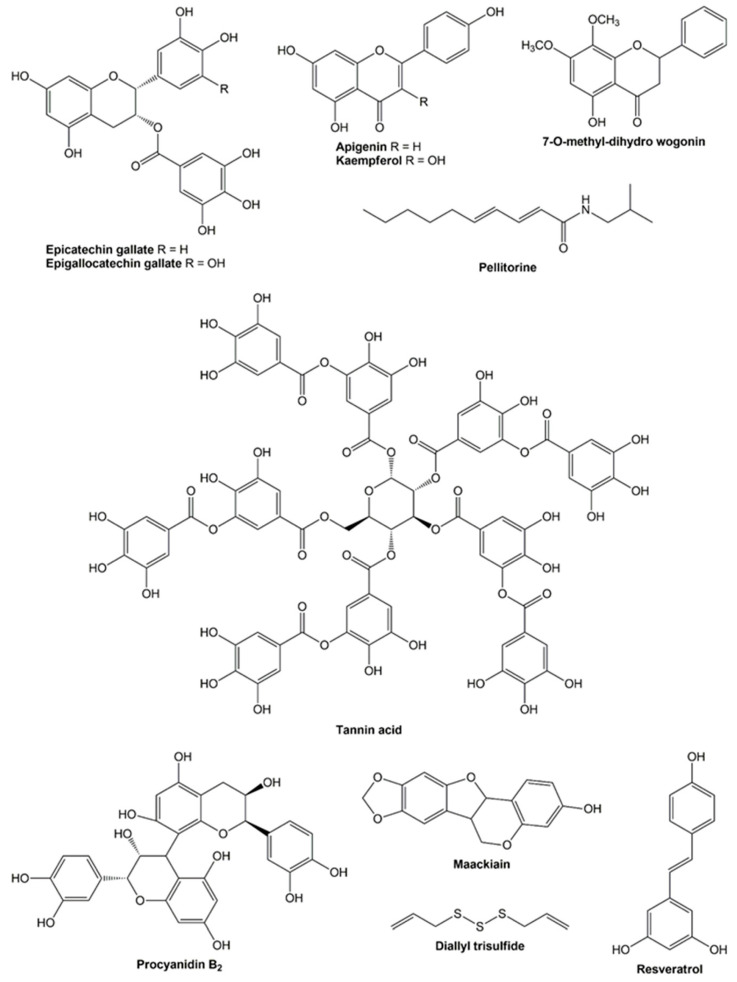
Chemical structures of the bioactive compounds.

As previously outlined, a variety of compounds have demonstrated a broad spectrum of properties effective in combating obesity. These bioactive compounds are derived from a variety of natural sources, including medicinal and edible plants, as well as seaweed. The anti-obesity effects of these compounds have been demonstrated to result from their inhibition of pancreatic lipase, in addition to their influence on the expression and secretion of various markers associated with different signaling pathways [44,61]. This review also underscores the anti-obesity potential of bioactive compounds when administered in conjunction with orlistat. Specifically, five flavonoids, two tannins, one pseudoalkaloid, one pterocarpan, one allyl sulfide, and one stilbenol have exhibited combined effects with the commercial pancreatic lipase inhibitor. A comprehensive overview of the properties is provided in Table 2. This table includes the biological models employed as well as the types of interactions between the compounds and orlistat. The chemical structures of these compounds are illustrated in Figure 2.

The majority of studies that have examined the potential of orlistat in conjunction with bioactive compounds have focused on its capacity to inhibit pancreatic lipase [70]. Flavonoids represent the most extensively studied chemical group. In this context, epicatechin gallate and epigallocatechin gallate have demonstrated synergistic effects on the inhibition of pancreatic lipase in combination with orlistat. These compounds have been identified in the methanol extract of leaves from *C. sinensis* (L.) Kuntze and its derived fractions, which have also demonstrated a synergistic inhibitory effect against pancreatic lipase when combined with orlistat. In this research, the synergistic potential of epicatechin gallate and/or epigallocatechin gallate was also observed after assessing various combinations of orlistat with these compounds. The CI values ranged from 0.27 to 0.71 for epicatechin gallate, from 0.26 to 0.62 for epigallocatechin gallate, and from 0.15 to 0.41 for a mixture of the two flavonoids and the commercially available pancreatic lipase inhibitor. The IC_50_ values were determined to be 0.70–3.00 for epicatechin gallate, 0.32–0.99 for epigallocatechin gallate, and 0.27–0.96 µM for a mixture of the two compounds and orlistat. The calculated IC_50_ values were lower than those for epicatechin gallate (IC_50_ = 12.75 µM) and epigallocatechin gallate (IC_50_ = 5.84 µM) evaluated individually [12].

Another flavonoid that has demonstrated a synergistic effect in inhibiting pancreatic lipase is kaempferol, a compound found in plants such as broccoli, cabbage, and tea. Kaempferol (0–229.20 μM) exhibited a significant synergistic effect when employed in conjunction with orlistat (0–60.48 μM). This enhancement in efficacy was observed to be concentration-dependent. When the concentrations of kaempferol and orlistat were below 114.60 and 30.24 μM, respectively, the CI value was less than 1, indicating synergistic inhibition between the two compounds [11]. Apigenin, a flavonoid found in various fruits, vegetables, and beans, has also demonstrated synergistic inhibitory effects against pancreatic lipase. Similar to kaempferol, apigenin (0–0.45 mM) exhibited a concentration-dependent inhibitory effect when combined with orlistat (0–60.48 μM). This observation was substantiated by a CI value of less than 1 when the concentrations of apigenin and orlistat were below 0.15 mM and 20.76 μM, respectively [76].

In another study, 7-O-methyl-dihydro wogonin exhibited a synergistic effect in inhibiting pancreatic lipase when used in conjunction with orlistat. This flavonoid, previously identified in the ethanol extract of roots from the medicinal plant *Andrographis paniculata* (Burm.f.) Nees, showed its synergistic properties at a concentration lower than 14.86 μM [77]. Conversely, the available evidence concerning the anti-lipase activity of compounds from other chemical classes in combination with orlistat is limited. A single study documented that pellitorine (a pseudoalkaloid identified in the ethanol extract of roots from *Piper longum*) exhibited a synergistic effect with orlistat (0.17 μM) at a concentration lower than 15.70 μM [78].

In addition to pancreatic lipase, cholesterol esterase represents another molecular target for the development of novel compounds to combat obesity. This enzyme is found in bile and functions by hydrolyzing dietary cholesterol esters and transporting free cholesterol from micelles to enterocytes. In addition, the inhibitory effect of polyphenols on cholesterol esterase activity has been well documented [83]. However, few natural products have been demonstrated to exert a combined inhibitory effect with orlistat. Procyanidin B_2_ and tannin acid are two tannins that have been identified in a variety of plants and their byproducts. The inhibitory potential of these compounds in conjunction with orlistat on cholesterol esterase was evaluated, and the resulting CI values were found to be less than 1, indicating that procyanidin B_2_ and tannin acid exhibited synergistic effects when used in combination with orlistat [79].

In contrast to the research conducted with pancreatic lipase or cholesterol esterase, there has been a paucity of studies determining the potential anti-obesity properties of bioactive compounds in combination with orlistat in other biological models or clinical trials. Maackiain, a pterocarpan identified in the roots from *Ononis spinosa* L., has been previously demonstrated to possess anti-adipogenic activity [84]. Consequently, the combination of this pterocarpan (100 μM) and orlistat (12 μM) was evaluated in *Caenorhabditis elegans*, a nematode model organism with functionally conserved genes involved in the modulation of lipid and carbohydrate metabolism. The authors observed that maackiain, when used in conjunction with orlistat, did not demonstrate toxicity and resulted in a decrease in lipid accumulation in *C. elegans*. However, the mixture only caused an increase in the mRNA expression of sbp-1 and the miRNA expression of miR-60. Consequently, given that the combination of maackiain and orlistat did not affect the expression of other markers related to lipid metabolism, the researchers concluded that this mixture did not present a synergistic interaction [80].

On the other hand, only one study was found that reported the combined potential of orlistat and a bioactive compound in an in vivo model. In this research, Wistar rats with induced obesity were administered orlistat (200 mg/kg) in conjunction with diallyl trisulfide (40 mg/kg), an allyl sulfide present in *A. sativum*. The combination of the compound and the commercial pancreatic lipase inhibitor resulted in a decrease in BW of the animals (<300 g), which was similar to the treatments with either diallyl trisulfide or orlistat. The combination of this drug and the allyl sulfide also caused a substantial reduction in serum glucose levels (<150 mg/dL), TG (<150 mg/dL), TC (<200 mg/dL), LDL cholesterol (<50 mg/dL), and VLDL cholesterol (<50 mg/dL). Furthermore, the combination resulted in a substantial decrease in serum liver function markers, including glutamate oxaloacetate transaminase (GOT, <75 IU/L), glutamate pyruvate transaminase (GPT, <60 IU/L), alkaline phosphatase (ALP, <160 IU/L), and LDH (<160 IU/L). These levels were found to be lower in comparison to the orlistat group [81].

Concurrent administration of diallyl trisulfide and orlistat resulted in increased levels of HDL cholesterol (>15 mg/dL) and markers associated with the antioxidant system in the liver and adipose tissue. These included catalase (CAT, >20 μmol H_2_O_2_ consumed/min/mg protein), glutathione peroxidase (GPx, >140 μmol glutathione oxidized/min/mg protein), reduced glutathione (GSH, >20 μg/mg protein), and SOD (>1.2 Units/mg protein). Conversely, the values observed in the group of animals treated with orlistat were significantly lower. In addition, the combination of diallyl trisulfide and orlistat led to a decrease in lipid peroxidation (LPO) levels in the liver and adipose tissue (<0.4 n moles of MDA formed/mg protein). This combination also resulted in a reduction of pathological alterations in these tissues (regeneration of specific areas in the liver and a decrease in fat cell accumulation). In contrast, the group treated with only orlistat exhibited a higher degree of these alterations [81].

Finally, resveratrol, a stilbenol found in various natural sources such as grapes, berries, and nuts, has also demonstrated anti-obesity properties in several animal models involving mice and rats, as well as clinical trials in patients suffering from obesity, metabolic syndrome, and other disorders [85]. However, a recent clinical study is the only one that has assessed the effects of this compound in combination with orlistat. In this research, obese patients were treated with a mixture consisting of orlistat (120 mg) and resveratrol (100 mg), which resulted in a significant reduction in BW (−3.31 kg), total body fat (TBF, −4.93%), and diastolic blood pressure (DBP, 75.93 mmHg). Conversely, subjects treated with only orlistat exhibited a reduction in BW of −2.92 kg, TBF of −0.75%, and DBP of 78.38 mmHg. A significant decrease in BW was also observed in patients with steatosis, fibrosis, and diabetes who received the combination of resveratrol and orlistat compared to those who received only orlistat. Furthermore, adverse events, including steatorrhea and diarrhea, were more prevalent in patients treated with orlistat alone. In contrast, steatorrhea was only observed in subjects treated with the combination of resveratrol and orlistat. The authors concluded that the combination of resveratrol and orlistat produces a synergistic interaction that leads to a reduction in BW and other beneficial effects observed in the study [82].

## 8. Conclusions and Future Perspectives

In this review, we have described the potential anti-obesity effects of various materials from natural sources and their bioactive compounds in combination with orlistat, a pancreatic lipase inhibitor. We observed that a variety of in vitro and in vivo models, along with clinical trials, were employed to assess the activity of combinations. A greater number of studies employing animal models, predominantly mice and rats, have substantiated the effectiveness of orlistat when utilized in conjunction with extracts, fractions, and other materials from natural sources. Subsequent to this, in vitro studies concentrating on pancreatic lipase and clinical studies were conducted. Conversely, a substantial body of in vitro studies has highlighted the inhibitory effects of bioactive compounds and orlistat on pancreatic lipase and cholesterol esterase, with fewer studies conducted on nematodes, rats, and patients with obesity.

However, further research is necessary to fully elucidate the role of combination therapy with orlistat and natural-derived materials or their compounds in the management of obesity. It is imperative that further research be conducted to ascertain the impact of extracts, fractions, and other natural-derived materials on pancreatic lipase and molecular markers such as cholesterol esterase. Additionally, it is crucial to evaluate whether these interactions with orlistat are synergistic, additive, or antagonistic. A substantial body of in vivo studies employing plant or seaweed samples has been identified. However, evidence for bioactive compounds is still limited to a single study. Consequently, it is imperative to identify and isolate the major compounds present in the extracts and other active natural-derived materials, as well as to determine their potential anti-obesity effects in combination with orlistat. It is also crucial to assess compounds with in vitro activity in animal models to ascertain their impact on lipid profiles and their potential to modify other markers associated with obesity, such as antioxidant enzymes and genes or proteins involved in signaling pathways like AMPK-PRDM16-UCP-1. It is fundamental to ascertain the non-toxicity of natural-derived materials (extracts, fractions, and other samples) and compounds in in vivo models prior to their administration in human trials.

It is crucial to evaluate the potential benefits of bioactive compounds on human health through clinical trials, especially in the absence of substantiating evidence. However, translating the results obtained into clinical applications is not straightforward, as a multitude of factors must be considered. These include verification of the reduction in adverse effects resulting from combined treatment with orlistat and the bioactive compound. Similarly, multi-target approaches must be explored, as combination therapy could have more than one mechanism of action. An additional element that warrants consideration pertains to formulation and administration, as a substantial proportion of the research has been directed towards single-active ingredient dosage forms. Consequently, there has been a paucity of studies conducted on the development of combination products, thereby hindering the ability to comprehend their pharmacokinetic and pharmacokinetic properties. Moreover, a compound may not readily combine with the drug, forming bulky substances that result in tablets that are difficult to swallow. Therefore, research is imperative for the co-formulation of one or more compounds with orlistat, enhancing the dissolution rate and solubility of the combined product. Finally, other factors that hinder the clinical implementation of a combination product pertain to socioeconomic dimensions, including the potential of the proposed combination therapy to enhance the nutritional status, quality of life, and safety of patients [86].

In addition to the aforementioned points, it is imperative that studies assess the same aspects prior to contemplating the utilization of natural sources and their bioactive compounds as a prospective combination therapy for obesity management. It is important to consider determining IC_50_ and CI values, inhibition percentages and mechanisms, and the type of interaction between orlistat and the sample of interest. The parameters evaluated should be reported on the same scales in in vitro assays, animal models, and clinical studies. This will ensure consistency with other works and serve as a reference for future research.

In conclusion, the available evidence suggests that natural sources and their bioactive compounds, in combination with orlistat, possess anti-obesity activity. The results obtained in various investigations are promising, establishing a starting point for further research that validates the efficacy and safety of combination therapy and considers it a therapeutic alternative in the management of obesity.

## Figures and Tables

**Table 1:** Anti-obesity effects of extracts and other natural-derived materials in combination with orlistat.

Natural Source	Biological Model	Effect	Combined Effect Interaction	Reference
*Camellia sinensis* L. Kuntze (extract of leaves and derived fractions)	Pancreatic lipase (in vitro)	Enzymatic inhibition (IC_50_ = 1.93–11.95 µg/mL)	Synergistic	[12]
*Ascophyllum nodosum* (L.) Le Jolis (tannin-rich fraction)	Pancreatic lipase (in vitro)	NA	Antagonistic	[63]
*Elettaria cardamomum* (extract of cardamom)	Mice with obesity (in vivo)	Decrease in BW, LOI, and blood glucose decrease in LDL (89 mg/dL), TC (83 mg/dL), TG (61.81 mg/dL), AI (0.33), and CRR (0.29) Positive effects on neuroinflammation and memory associated with obesity	Synergistic	[64]
*Gelidium elegans* (extract of the edible seaweed)	Mice with obesity (in vivo)	Decrease in BW gain and percentage of BW (subcutaneous fat) Decrease in serum TG (<60 mg/dL), hepatic TG (<125 mg/g liver), serum insulin (<5 ng/mL), and blood glucose (116.7–156.0 mg/dL) Increase in HDL (109.6 mg/dL) White adipose tissue: decrease in protein expression of C/EBPα (<90%) and PPARγ (<90%) Hepatic tissue: increase in AMPK phosphorylation (>175%) and PRDM16 (>130%); decrease in protein expression of SREBP-1 (<80%), ACC (<40%), DGAT-1 (<60%), and FAS (<30%) Brown adipose tissue: increase in AMPK phosphorylation (>225%) and protein expression of PRDM16 (>150%) and UCP-1 (>130%) Decrease in hepatic lipogenesis (histopathological assays) and liver weights (1.4 g)	Synergistic	[65]
*Hibiscus tiliaceus* L. (extract of leaves)	Rats with obesity (in vivo)	Decrease in BW (174.44 g), TG (166.92 mg/dL), and TC (188.33 mg/dL) Increase in HDL (67.84 mg/dL)	NS	[66]
*Allium sativum* (extract of cloves)	Rats with obesity (in vivo)	Decrease in feed intake (<150 g), BW (<300 g), free fatty acids (<100 µmol/L), glucose (<125 mg/dL), insulin (<100 µIU/mL), HOMA-IR index (<20), and leptin (<4 ng/mL) Increase in ghrelin (>0.3 Pg/mL) and adiponectin (>20)	NS	[67]
*Cichorium intybus* L., *Foeniculum vulgare* Mill., and *Solanum nigrum* L. (aqueous distillates of the plants)	Rats with obesity (in vivo)	Decrease in BW gain (<30 g), FER (<0.075), BMI (<0.75 gm/cm^2^), HW:BW ratio (<4), and RLW (<7.5) Decrease in TC (<110 mg/dL), TG (<110 mg/dL), LDL (<75 mg/dL), lipase (<200 U/L), glucose (<125 mg/dL), insulin (<1.25 µg/mL), leptin (<2.5 ng/mL), LDH (<125 IU/L), TBARS (<75 nmol/g tissue protein), and AI (<3) Increase in HDL (>30 mg/dL) and SOD (>8 Units/mg protein) Normal histology in liver and heart	NS	[68]
*Garcinia cambogia* (capsules)	Obese patients (clinical study)	Decrease in BW (83.22 kg), BMI (30.45 kg/m^2^), WC (84.16 cm), HC (102.17 cm), VAI (2.17), SBP (133.10 mmHg), PP (52.97 mmHg), MBP (106.61 mmHg), TG (221.92 mg/dL), TC (187.31 mg/dL), VLDL (44.38 mg/dL), CRR (3.81), and postprandial blood glucose (131.17 mg/dL) Increase in HDL (49.11 mg/dL)	Additive	[69]
*Plantago ovata* (psyllium)	Obese patients (clinical study)	Decrease in BW (94.96 kg) and BMI (37.40 kg/m^2^) Decrease in the number of gastrointestinal events (oily spotting = 42, fecal urgency = 41, and incontinence = 11)	NS	[70]

NA: not applicable (the sample exhibited no activity in conjunction with orlistat). NS: not specified (the article did not specify whether there is a combined effect interaction). ACC: acetyl-CoA carboxylase. AI: atherogenic index. AMPK: adenosine monophosphate-activated protein kinase. BMI: body mass index. BW: body weight. C/EBPα: CCAAT/enhancer binding protein α. CRR: cardiac risk ratio. DGAT-1: diacylglycerol O-acyltransferase-1. FAS: fatty acid synthase. FER: food efficiency ratio. HC: hip circumference. HDL: high-density lipoprotein. HOMA-IR: homeostasis model assessment of insulin resistance. HW: heart weight. IC_50_: mean inhibitory concentration. LDH: lactate dehydrogenase. LDL: low-density lipoprotein. LOI: Lee obesity index. MBP: mean blood pressure. PP: pulse pressure. PPARγ: peroxisome proliferator-activated receptor γ. PRDM16: PR domain-containing16. RLW: relative liver weight. SBP: systolic blood pressure. SOD: superoxide dismutase. SREBP-1: sterol regulatory element-binding protein-1. TBARS: thiobarbituric acid-reactive substances. TC: total cholesterol. TG: triglycerides. UCP-1: uncoupling protein-1. VAI: visceral adiposity index. VLDL: very low-density lipoprotein. WC: waist circumference.

**Table 2:** Anti-obesity effects of compounds in combination with orlistat.

Compound	Biological Model	Effect	Combined Effect Interaction	Reference
Flavonoids				
Epicatechin gallate and/or epigallocatechin gallate	Pancreatic lipase (in vitro)	Enzymatic inhibition by epicatechin gallate (IC_50_ = 0.70–3.00 µg/mL), epigallocatechin gallate (IC_50_ = 0.32–0.99 µg/mL), and a mixture of the compounds (IC_50_ = 0.27–0.96 µg/mL)	Synergistic	[12]
Kaempferol	Pancreatic lipase (in vitro)	Enzymatic inhibition (concentrations < 114.60 μM)	Synergistic	[11]
Apigenin	Pancreatic lipase (in vitro)	Enzymatic inhibition (concentrations < 0.15 mM)	Synergistic	[76]
7-O-methyl-dihydro wogonin	Pancreatic lipase (in vitro)	Enzymatic inhibition (concentrations < 14.86 μM)	Synergistic	[77]
Pseudoalkaloid				
Pellitorine	Pancreatic lipase (in vitro)	Enzymatic inhibition (concentrations < 15.70 μM)	Synergistic	[78]
Tannins				
Procyanidin B_2_	Cholesterol esterase (in vitro)	Enzymatic inhibition	Synergistic	[79]
Tannin acid	Cholesterol esterase (in vitro)	Enzymatic inhibition	Synergistic	[79]
Pterocarpan				
Maackiain	Nematodes (*Caenorhabditis elegans*)	Decrease in lipid accumulation Increase in mRNA expression of sbp-1 and miRNA expression of miR-60	NA	[80]
Allyl sulfide				
Diallyl trisulfide	Rats with obesity (in vivo)	Decrease in BW (<300 g) Decrease in glucose (<150 mg/dL), TG (<150 mg/dL), TC (<200 mg/dL), LDL (<50 mg/dL), VLDL (<50 mg/dL), GOT (<75 IU/L), GPT (<60 IU/L), ALP (<160 IU/L), and LDH (<160 IU/L) Increase in HDL (>15 mg/dL) Liver and adipose tissue: decrease in LPO (<0.4 n moles of MDA formed/mg protein) Liver and adipose tissue: increase in CAT (>20 μmol H_2_O_2_ consumed/min/mg protein), GPx (>140 μmol glutathione oxidized/min/mg protein), GSH (>20 μg/mg protein), and SOD (>1.2 Units/mg protein) Decrease in alterations in liver and adipose tissue (histopathological assays)	NS	[81]
Stilbenol				
Resveratrol	Obese patients (clinical study)	Decrease in BW (−3.31 kg), TBF (−4.93%), and DBP (75.93 mmHg) Decrease in BW in patients with steatosis, fibrosis, and diabetes Steatorrhea as an adverse effect	Synergistic	[82]

NA: not applicable (the compound did not demonstrate a combined effect interaction). NS: not specified (the article did not specify whether there is a combined effect interaction). ALP: alkaline phosphatase. BW: body weight. CAT: catalase. DBP: diastolic blood pressure. GOT: glutamate oxaloacetate transaminase. GPT: glutamate pyruvate transaminase. GPx: glutathione peroxidase. GSH: reduced glutathione. HDL: high-density lipoprotein. IC_50_: mean inhibitory concentration. LDH: lactate dehydrogenase. LDL: low-density lipoprotein. LPO: lipid peroxidation. SOD: superoxide dismutase. TBF: total body fat. TC: total cholesterol. TG: triglycerides. VLDL: very low-density lipoprotein.

## Data Availability

No new data were created.

## Abbreviations

The following abbreviations are used in this manuscript:

ACC	Acetyl-CoA carboxylase
AI	Atherogenic index
ALP	Alkaline phosphatase
AMPK	Adenosine monophosphate-activated protein kinase
AUC	Area under the curve
BP	Blood pressure
BMI	Body mass index
BW	Body weight
C/EBPα	CCAAT/enhancer binding protein α
CAT	Catalase
CI	Combination index
CRR	Cardiac risk ratio
DBP	Diastolic blood pressure
DGAT-1	Diacylglycerol O-acyltransferase-1
FAS	Fatty acid synthase
FER	Food efficiency ratio
GOT	Glutamate oxaloacetate transaminase
GPT	Glutamate pyruvate transaminase
GPx	Glutathione peroxidase
GSH	Reduced glutathione
HC	Hip circumference
HDL	High-density lipoprotein
HPLC	High-performance liquid chromatography
HOMA-IR	Homeostasis model assessment of insulin resistance
HW	Heart weight
IC_50_	Mean inhibitory concentration
LDH	Lactate dehydrogenase
LDL	Low-density lipoprotein
LOI	Lee obesity index
LPO	Lipid peroxidation
MBP	Mean blood pressure
NA	Not applicable
NF-κB	Nuclear factor κB
NS	Not specified
PP	Pulse pressure
PPAR	Peroxisome proliferator-activated receptor
PRDM16	PR domain-containing 16
RLW	Relative liver weight
SBP	Systolic blood pressure
SOD	Superoxide dismutase
SREBP-1	Sterol regulatory element-binding protein-1
TBARS	Thiobarbituric acid-reactive substances
TBF	Total body fat
TC	Total cholesterol
TG	Triglycerides
UCP-1	Uncoupling protein-1
VAI	Visceral adiposity index
VLDL	Very low-density lipoprotein
WC	Waist circumference
WHO	World Health Organization
